# A highly complex variant of the plantaris tendon insertion and its potential clinical relevance

**DOI:** 10.1007/s12565-020-00540-4

**Published:** 2020-04-04

**Authors:** K. Kurtys, B. Gonera, Ł. Olewnik, P. Karauda, M. Polguj

**Affiliations:** 1 Department of Anatomical Dissection and Donation, Medical University of Lodz, Żeligowskiego 7/9, 90-136 Łódź, Poland; 2 Department of Normal and Clinical Anatomy, Medical University of Lodz, Żeligowskiego 7/9, 90-136 Łódź, Poland

**Keywords:** Achilles tendinopathy, Achilles tendon, Anatomical variations, Calcaneal tendon, Case report, Plantaris muscle, Plantaris tendon

## Abstract

The body is home to a number of unique and intriguing anatomical structures, plenty of which concern the muscles and their tendons. Of these, the plantaris muscle is reported to present a particularly high range of morphological variations. The muscle, passing distally throughout the length of the lower leg, consists of a small muscle belly and a long, thin tendon. It originates, traditionally, on the popliteal surface of the femur and the knee joint capsule, and then inserts to the calcaneal tuberosity. It has been suggested that mid-portion Achilles tendinopathy may be caused by certain plantaris tendon morphologies. This case report describes a new anomalous plantaris tendon insertion, closely related to the Achilles tendon. It comprise four distinct insertions and one direct merge with the calcaneal tendon. The current classification should be extended to accommodate such ‘rare cases’ to facilitate more successful Achilles tendinopathy treatment.

## Introduction

A human body conceals many enigmatic anatomical structures which are frequently depicted in case studies. Although some are considered to be more spectacular and clinically significant than others, all are unique and merit our attention.

Anatomical variations can be observed around nerves, veins, arteries and ligaments, as well as muscles and their tendons (Tubbs et al. 2009; Sugavasi 2013; Polguj et al. 2014; Angelov and Jelev 2016; Olewnik et al. 2017a, b, 2018a, b, 2019a, b, c; Terfera et al. 2015; Xu et al. 2019; Piagkou et al. 2019). Some of these structures have a tendency to display more common variations, and these can be used to create formal classification systems (Tubbs et al. 2009; Olewnik et al. 2017b, 2018a, 2019a, b, c). Such findings are of great value for surgical procedures in medical disciplines, such as orthopaedics, vessel surgery or neurosurgery (Tubbs et al. 2009; Olewnik et al. 2018a, 2019a, c). However, remarkably rare anatomic anomalies that do not fit the classification have also been observed, and these should not be ignored because of their possible influence on the health of patients.

The plantaris muscle (PM) is one such structures. This muscle is characterized by a small, slim and spindle-shaped belly located within the superficial posterior compartment of the leg, and which originates casually on the popliteal surface of the femur, superior to the lateral condyle of the femur and the knee joint capsule. The belly then develops into a long, thin tendon which heads toward and inserts on the calcaneal tuberosity, after passing through the space between the gastrocnemius muscle and the soleus muscle. Typically, innervation is provided by muscular branches of the tibial nerve and vascularity by muscular branches of the popliteal artery.

The PM is believed to be one of the vestigial muscles of the body (Cruveilhier 1834). A few studies have reported that its prevalence ranges from 81.8 to 96% (Harvey et al. 1983; Simpson et al. 1991; Freeman et al. 2008; Nayak et al. 2010; Olewnik et al. 2017b, 2018b), but two suggest that it could be present in 100% (Aragão et al. 2010; van Sterkenburg et al. 2011a). It demonstrates anatomical variations in both its proximal and distal attachments, as well as in its course (Ahmed et al. 2017; Cummins and Anson 1946; van Sterkenburg et al. 2011a; Olewnik et al. 2017b) and sporadic, individual variations have also been described (Upasna and Kumar 2011; Sugavasi 2013; Srimani et al. 2014; Olewnik et al. 2017a).

Interestingly, it has been suggested that the course of the plantaris tendon (PT) influences the etiopathogenesis of Achilles tendinopathy and pain in the medial crural region (van Sterkenburg et al. 2011a; Alfredson 2011; Spang et al. 2013; Ballal et al. 2014; Mao et al. 2015; Alfredson and Spang 2017). Moreover, its long tendon appears ideal for harvesting for the use in reconstructions of other tendinous and ligamentous structures (Simpson et al. 1991; Pagenstert et al. 2005; Kotian et al. 2013).

This study describes a rare case of the PT insertion, and its findings serve as a valuable complement to existing classifications of PT insertion. A better understanding of the relationship between the PT and the Achilles tendon (AT) may be precious for scientists investigating the participation of the PT in etiopathogenesis of Achilles tendinopathy.

## Case report

The cadaver of a 68-year-old male was subjected to routine anatomical dissection at the Department of Normal and Clinical Anatomy, Medical University of Lodz for the purposes of research and the education of medical students. The dissection of the knee and crural region and foot area was performed using standard techniques according to a strictly specified protocol (Olewnik et al. 2018a, 2019a, b).

During dissection, the PM belly was recognized, found to be normal and cleared. Its origin was located on the knee joint capsule under the lateral head of the gastrocnemius muscle. No abnormalities concerning the muscle belly were detected. The long, thin PT was then subjected to exhaustive deterging by eye. The PT ran downwards between the soleus muscle and the medial head of the gastrocnemius, after which, it appeared medial to the calcaneal tendon. The course thus far was typical and no anomalies were distinguished. Following this, the PT was found to run close to the AT. At a point half way along the AT, the first furcation was observed: the main tendon forked off two thinner bands (A, B) which descended parallel to each other and subsequently diverged again. The band A divided (A1, A2) 18.03 cm above the calcaneal tuberosity and the band B (B1, B2) 16.25 mm higher. All additional connections, bands and their attachments were cleansed and strictly examined:

- Band A1 was characterized by a fan-shaped insertion. Its attachment was located on the superomedial surface of the calcaneal tuberosity separately from the AT.
- Band A2 attached to the medial surface of the calcaneus body as a straight and plain band.
- Band B1 inserted to the superior surface of the calcaneus, anterior to the calcaneal tendon nearby its medial ridge.
- Band B2, the last of the distal attachments, inserted to the medial surface of the calcaneus body, medial and inferior to B1 and anterior to A2.

**Fig. 1:**
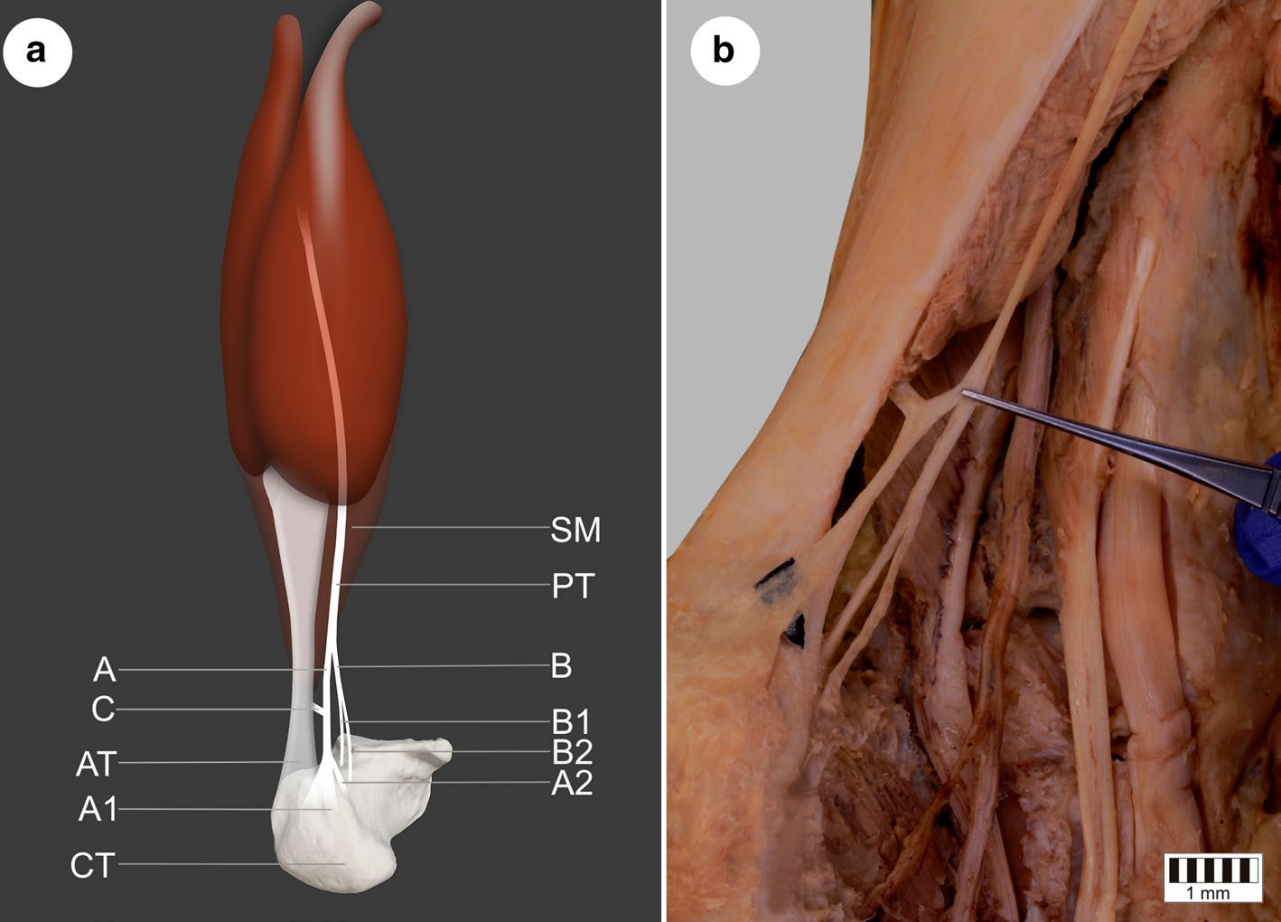
**a** The presented variant of the plantaris tendon insertion: a posteromedial view of the left crural and foot region. **b** The Type VII of plantaris tendon insertion; *SM* soleus muscle, *PT* plantaris tendon, *AT* Achilles tendon, *CT* calcaneal tuberosity, *A, A1, A2, B, B1, B2, C* specific bands of the plantaris tendon (Type VII); the solid line surrounds the superior surface of the calcaneus; the dashed line surrounds the medial surface of the calcaneus

**Fig. 2:**
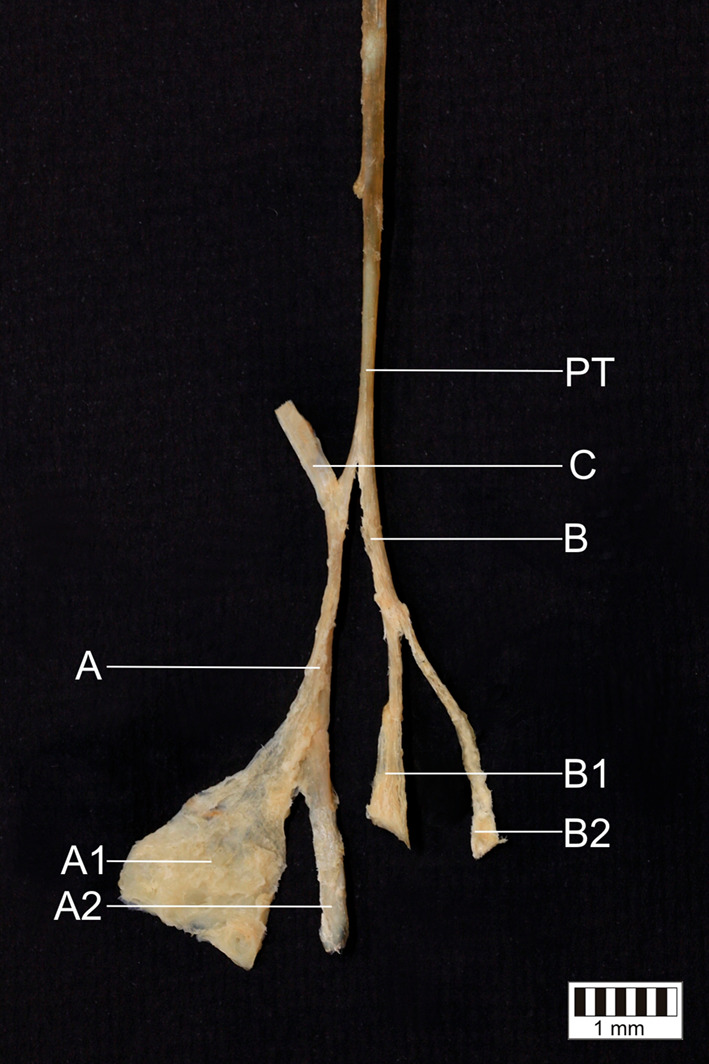
The presented Type VII plantaris tendon insertion after removal of attachments. *PT* plantaris tendon, *A, A1, A2, B, B1, B2, C* specific bands of the plantaris tendon (Type VII)

Additionally, a specific tendinous bridge (band C) was distinguished between band A and the AT. It originated on the first centimetre of band A, and winds toward the anteromedial surface of the AT (Figs. 1, 2).

Appropriate morphometric measurements were acquired. These were taken as digital photographic images and processed through MultiScanBase 18.03 (Computer Scanning System II, Warsaw, Poland). All results are presented in Table 1.

**Table 1:** A presentation of morphometric measurements of all bands

Band	*A*			*B*	
Altitude of the diverging measured from the calcaneal tubercle (mm)	18.03			34.28	
Length (mm)	32.68			17.18	
Width in the middle point (mm)	1.48			1.57	
Band	A1	A2	C	B1	B2
Length (mm)	16.93	16.36	10.24	19.02	23.98
Width in the insertion point (mm)	15.35	2.21	1.93	3.32	2.66
Altitude of the insertion measured from the calcaneal tubercle (mm)	–	–	44.01	–	–

## Discussion

The PM has been a popular subject of recent anatomical studies relevant to the pelvic limb, especially the lower leg (Ahmed et al. 2017; Aragão et al. 2010; Nayak et al. 2010; van Sterkenburg et al. 2011a; Olewnik et al. 2017b, 2018b). Despite this, many aspects of the PM remain unclear including its true function or relationship with the Achilles tendinopathy, what implies that further research is still needed (Menton 2000; van Sterkenburg et al. 2011a; Olewnik et al. 2017b, 2018b; Vlaic et al. 2019). It is often regarded as a vestigial and useless muscle in evolutionary science because of its small role in biomechanics (Menton 2000). According to Cruveilhier’s theory, the PT lost its primary insertion into the plantar aponeurosis over the course of evolution, and consequently attached to the calcaneal tuberosity (Cruveilhier 1834). It is also reported to have been a primitive flexor of the toes (Daseler and Anson 1943). In addition, the presence of a small muscle belly, a long, thin tendon, and a high density of muscle spindles suggest that the PM may be a highly-specialized *sensory* muscle rather than *motor* one (Menton 2000). The anatomical, evolutional and biomechanical misconceptions regarding the PM demand further study, and our present findings shed new light on the variability of the PT insertion.

Various classification systems have been created for the PT (Cummins and Anson 1946; van Sterkenburg et al. 2011a; Olewnik et al. 2017b). In 1946, Cummins et al. (1946) presented a fourfold classification of PT insertion identified during dissection of 200 lower limbs in the crural and foot region. Sterkenburg et al. (2011a) later reported the PT to demonstrate as many as nine different insertion variants. Two recent studies published by Olewnik et al. (2017b,2018b) contain a description of six types of insertion, with the PM being present in 96% of cases in the former, and 89.2% in the latter. A fuller classification and the prevalence of each type (Olewnik et al. 2017b, 2018b) is given in Table 2. Moreover, one of these two studies describe two variants (A and B) based on the relationship between the course of the PT and the AT. The more common variant A (84%) was found to pass through the space between the gastrocnemius muscle and the soleus muscle and descend along the medial part of the lower leg, medial to the AT. The other (B) was determined by a slightly different course: after crossing the space between the gastrocnemius and the soleus muscle and running down the medial part of the lower leg, the PT descended anterior to the calcaneal tendon (Olewnik et al. 2017b). The PT identified in the present study was found to follow variant A, but it did not display any of the PT insertion types described in previous studies.

**Table 2:** The classifications of the plantaris tendon insertion

Type of the PT insertion	Olewnik et al. (2017a; b) (%)	Olewnik et al. (2018a; b) (%)	The presenting study
Type 1—the wide, fan-shaped insertion into the calcaneal tuberosity on the medial side of the Achilles tendon	44	44	–
Type 2—the calcaneal tuberosity on the medial side, along with the Achilles tendon	18	22.4	–
Type 3—the calcaneus, anterior to the Achilles tendon	8	6.9	–
Type 4—the deep crural fascia	4	3.4	–
Type 5—wide insertion encircling the posteromedial surface of the Achilles tendon	22	18.1	–
Type 6—the point nearby the tarsal canal flexor retinaculum of the leg	–	5.2	–
Type 7—‘rare cases’:	–	–	1 Case
The A1 band—fan-shaped insertion on the superomedial surface of the calcaneal tuberosity			
The A2 band—the medial surface of the calcaneus body			
The B1 band—the superior surface of the calcaneus, anterior to the Achilles tendon nearby its medial ridge			
The B2 band—the medial surface of the calcaneus body			
The C band—the anteromedial surface of the Achilles tendon			

We propose the further extension of the PT insertion classification system, which currently includes six types (Olewnik et al. 2017b, 2018b) (Table 2). One of the two following terms should be included as Type VII—‘rare cases’:

- Diverged insertion—at least two distinct insertions;
- Direct, tendinous connection with the AT—located above the distal attachment.

The variant of the PT insertion presented in the present study can be categorized as Type VII based on either term (Figs. 1, 2).

In addition to the classified variations of the PT based on its insertion and course, some extremely rare cases have been described (Upasna and Kumar 2011; Sugavasi 2013; Srimani et al. 2014; Olewnik et al. 2017a). Two of them differ in the origin of the PM, with two separate muscle bellies developing into a common tendon (Upasna and Kumar 2011; Srimani et al. 2014). Cases of an absent PM have also been published (Harvey et al. 1983; Simpson et al. 1991). Additionally, another report describes the presence of a small muscle belly connected around the origin of the soleus muscle by thin fascia; however, no mention was made of the PT identified in the present study (Sugavasi 2013). Another one reports a rare case of the co-occurrence of a PM distal attachment within the right leg and its absence within the left leg; in this case, the PT insertion was located in the deep fascia of the leg (Olewnik et al. 2017a). However, the distal part of any PT did not appear to be as elaborated as in the present case.

Four distinct distal attachments were identified in the present study (A1, A2, B1, B2) (Figs. 1, 2). The insertion of band A1 resembles the Type V insertion proposed by Olewnik et al. (2017b): i.e. attaching with the broad, fan-shaped insertion to the superomedial surface of the calcaneal tuberosity, but there were no specific connection with the AT. Band A2 displays a strong and plain insertion into the medial surface of the calcaneus, located medial and anterior to band A1. Both bands B1 and B2 descend parallel to each other; B1 inserts to the superior surface of the calcaneus, anterior to the AT, while B2 attaches to the medial surface of the calcaneus, anterior to A2. Band B2 has the most medial position and appears to be the weakest of the bands. It not only consists of a multiple distal attachment complex, but also forms a direct connection with the anteromedial surface of the mid-portion of the AT (band C). More precisely, it is located close to the place where the soleus muscle becomes the AT (Fig. 1). No previous study has depicted a similar kind of tendinous merge between these two tendons.

Despite its small size, the PM seems to be clinically significant in some medical issues (Freeman et al. 2008; van Sterkenburg et al. 2011a, b; Alfredson 2011; Rohilla et al. 2013; Spang et al. 2013; Alfredson and Spang 2017; Olewnik et al. 2018b; Vlaic et al. 2019). As it has little importance in lower limb movements and possesses a long, thin tendon, it is considered appropriate grafting material for reconstructions of other anatomical structures, such as the anterior talofibular ligament, the calcaneofibular ligament and some of the muscle tendons of the upper limb (Simpson et al. 1991; Pagenstert et al. 2005; Kotian et al. 2013). The type of the tendon examined in our present case may serve as a source of confusion during harvesting procedures because of its complexity. Furthermore, rupture of the PM can imitate deep vein thrombosis, and the two conditions require differentiation by ultrasound examination (Rohilla et al. 2013).

Tendinopathy of the calcaneal tendon is a difficult disorder to treat (van Sterkenburg et al. 2011a, b; Alfredson 2011; Spang et al. 2013; Olewnik et al. 2017a, b; Alfredson and Spang 2017), typically due to the miscomprehension of its etiopathogenesis. One of its recognized symptoms is the presence of chronic pain within the calcaneal tendon. It is typically associated with athletes, but it can also arise in inactive patients.

In addition, Achilles tendinopathy has also been reported to be influenced by the course of the PT (van Sterkenburg et al. 2011a; Alfredson 2011; Spang et al. 2013; Ballal et al. 2014; Mao et al. 2015; Alfredson and Spang 2017). The closest relationship between the PT and the AT was observed at the level of the mid-portion of the AT (van Sterkenburg et al. 2011b). According to Alfredson et al. (Alfredson 2011), in the majority of patients, the pain related to this issue was located 2–7 cm above the calcaneal tuberosity, on the medial side. It has to be emphasized that a direct merge between the presenting PT and AT was identified at an altitude of 44.01 mm (the mid-portion) above the calcaneal tuberosity and anteromedial to the AT. Hence, it is crucial for clinicians to be more aware of the possible variations of the PT, and be prepared to identify such ‘rare’ connections during the diagnosis and treatment of Achilles tendinopathy.

Although the presented case was examined reliably and describes an attractive anatomical anomaly, it does include few limitations. The proposal that the course of the PT may cause the tendinopathy of the calcaneal tendon is only based on the assumptions and speculation derived from the previous studies. Further and more clinical studies are needed to identify such cases with a direct connection between the two tendons and subject them to deeper analysis. Nevertheless, our findings may serve as a base for future studies by indicating that such merges are possible.

## Conclusion

Our study illustrates the high tendency to variability of the plantaris tendon by describing a highly complex insertion comprising four distinct attachments. It is proposed as a new type VII—‘rare cases’ the plantaris muscle distal attachment and demonstrates a direct, tendinous connection with the Achilles tendon. Considering such intricacy and its relationship with the calcaneal tendon, it may influence the development of mid-portion Achilles tendinopathy and represent a source of confusion during harvesting procedures. Such knowledge may be of value for clinicians treating Achilles tendinopathy and scientists researching the relationship between this medical problem and the plantaris tendon.
